# How accurate are runners’ prospective predictions of their race times?

**DOI:** 10.1371/journal.pone.0200744

**Published:** 2018-08-01

**Authors:** Konstantinos Liverakos, Kate McIntosh, Christopher J. A. Moulin, Akira R. O’Connor

**Affiliations:** 1 School of Psychology & Neuroscience, University of St Andrews, St Andrews, Fife, Scotland, United Kingdom; 2 Laboratoire de Psychologie et Neurocognition, CNRS 5105, Universite Grenoble Alpes, Grenoble, France; National Center of Medicine and Science in Sport, TUNISIA

## Abstract

Metacognition is a domain which has illuminated our understanding of the regulation of cognition, but has yet to be applied in detail to more physical activities. We used half marathon finish time predictions from 7211 runners to investigate the factors that influence running performance metacognitive accuracy. In particular, we were concerned with the effects of experience, gender, and age on calibration. We expected more experienced runners to be better calibrated than less experienced ones. Given analogous findings in the domain of metacognition, we expected women to be less overconfident in their predictions, and better calibrated than male runners. Based on the metacognition literature, we expected that if older runners have effectively learned from previous experience, they would be as well-calibrated as younger runners. In contrast, uninformed inferences not based on performance feedback would lead to overestimating performance for older compared to younger runners. As expected, experience in terms of both club membership and previous race completion improved calibration. Unexpectedly though, females were more overconfident than males, overestimating their performance and demonstrating poorer calibration. A positive relationship was observed between age and prediction accuracy, with older runners showing better calibration. The present study demonstrates that data, collected before a test of physical activity, can inform our understanding of how participants anticipate their performance, and how this ability is affected by a number of demographic and situational variables. Athletes and coaches alike should be aware of these variables to better understand, organise, plan, and predict running performance, potentially leading to more appropriate training sessions and faster race finish times.

## Introduction

An issue of critical importance for many who engage in physical activity is how well they will perform the activity in question. Performance predictions become more important the more seriously the athlete takes their sport: goals are set on the basis of an awareness of current abilities and expected outcomes, and training is modified according to feedback from actual performance in comparison to these goals [1]. As such, inability to set realistic will lead to sub-optimal performance, including failing to train adequately, starting a race too fast, or not being able to set realistic goals which improve confidence and motivation.

Although metacognitive approaches to sport exist—for a review, see [2]—there has been little or no research on operationalising measures of metacognition made by competitors. Whilst considerable research has been conducted on predicting performance using training measures, body composition, and experience based on previous personal best times [3–10], much less effort has been expended on understanding how accurate runners *themselves* are in predicting their own performance. We therefore investigated the accuracy of runners’ predictions of their own race times. We drew on a larger body of literature that considers metacognitive predictions of cognitive performance. This literature pointed us to a series of benchmarks in performance prediction, such as the effects of experience, age, gender, and practice. We briefly review these effects below, before outlining our hypotheses regarding how these metacognition effects may translate into running performance prediction effects in the current data.

The metacognitive system is most often operationalised as feedback between two levels: the object level, which comprises the current operations, and the meta level, an abstract, higher-level representation of the object level which includes expectations and beliefs [11]. Between these two levels of function and representation, there are reciprocal flows of information. In metacognitively *monitoring* the object level, we reflect on our performance in comparison to our goals (usually measured by making explicit predictions of performance). In metacognitively *controlling* the object level, we exert influence on it. A critical debate in metacognition relates to how metacognitive decisions are made, and this pertains to whether they are made prospectively, before a task, or retrospectively, after having completed a task [12]. In general, prospective evaluations are less accurate than retrospective evaluations [13], mostly because prospective judgements are not party to the same task-specific information based on experience and feedback. The focus of this study was prospective evaluations: being able to make an accurate prospective judgement is most likely to be critical in how we adapt to and prepare for a task.

The capacity to accurately metacognitively predict future performance is termed calibration, i.e. calibration represents the capacity to make predictions which are of a similar or identical magnitude to actual performance [14]. Being well calibrated can aid cognitive processes such as learning; performance-related calibration should aid athletes who wish to understand their own performance [15–18]. According to a metacognitive account, prospective judgements that are poorly calibrated would lead to overconfident athletes, who would (for example) likely be disappointed after races, compared with well-calibrated runners who accurately predict their finish times. Understanding how potential factors such as calibration influence motivation, adherence to training regimens and ultimately performance, has the potential to impact on many levels, from public health programmes aiming to increase participation [19–21] through to elite achievement [22]. Before we can understand these influences however, we must first gauge whether concepts such as calibration are applicable to athletic performance. Metacognitive calibration is influenced by a number of factors, including experience, gender and age. Looking across both performance and cognitive literatures, we review these factors below, with a view to interrogating their effects on performance calibration in our running sample.

Research using cognitive tasks has demonstrated a significant positive effect of experience on metacognitive processes. Kratzig and Arbuthnott [23] found that item-specific experience (i.e. task repetition) increased metacognition calibration in a memory task. Similarly, Brown, Smiley and Lawton [24] demonstrated beneficial effects on planning and strategy for cognitive task completion.

Most studies which have considered experience and calibration in sport have focused on complex procedural processes within golf and tennis [16, 17], making them difficult to compare to the straightforward, consciously available processes assessed during metacognitive evaluation. In this study, a measure of experience was drawn from the demographic data provided at registration—running club membership. We assumed that runners who have trained with a club would be more likely to both be more experienced, and have access to more extensive feedback in terms of coaching and advice from fellow runners. We therefore predicted that runners with a running club affiliation would make more accurate predictions of their times than unaffiliated runners. Another, more direct marker of experience is the number of times a runner completed the same race over the years for which we had data (from 2009 to 2015, excluding 2010). We anticipated that runners with multiple participation records would be better calibrated at later compared to earlier races.

Gender differences are widely observed in the use of both planning and monitoring behaviours relating to cognition. Topçu and Yılmaz-Tüzün [25] found that females aged 9 to 14 had better knowledge of their cognition and were better able to regulate it than males. Similarly, Bidjerano [26] reported that undergraduate female students scored higher than male students on the metacognition subscale of a standardised education questionnaire. These findings suggest that women are more likely to better monitor performance in academic domains. Gender effects on calibration, which result from men displaying inappropriately high confidence, have also been consistently reported. For example, Lundeberg, Fox and Punćcohaŕ [27] found undergraduate males who had given an incorrect answer in lab course tests on various academic domains (e.g. computational skills and descriptive statistics) to be more overconfident than females in the same situation. Interestingly, their data suggested that both females and males were overconfident relative to their actual performance, but with men more so than women.

Relevant to sport, women are more aware of their body composition. Knechtle, Rosemann, Knechtle, and Bescos [28] investigated gender differences in Basic Metabolic Index (BMI) estimates collected before races in different sports between 2006 and 2011. Men were more than twice as likely to misjudge their BMI compared to women (12.4% vs. 5.2%). Men were also more likely to indicate their BMI to be within the healthy range when objective measurements placed them in the overweight category. Taken together, the aforementioned findings suggest a general tendency of men to be overconfident in measures of cognition and health, whilst women are more likely to reflect accurately on these measures. Therefore, it would similarly be expected that male runners in the present study would be more likely to predict faster-than-completed finish times, whilst female runners should be more accurate in their predictions.

To our knowledge, there is no published research on the effects of age on performance prediction in sports, even though it is a critical factor in race performance itself [29, 30]. There is however, a well-developed literature on the effects of age on metacognitive evaluations, especially in memory. There are mixed findings regarding metacognitive age effects, with some studies suggesting that the accuracy metacognitive evaluations is impaired by the aging process, and others that there are no age-related changes. Interestingly, given the effects of experience, to our knowledge there are no studies that show older adults make more accurate judgements on cognitive tasks, but this could be due to a lack of transfer between expertise gained in daily life and the types of tasks used to measure cognitive function. (Equally it could be due a failure to remember recent experiences or update representations of performance.) In general, older adults make appropriate retrospective judgements in metacognition. For example, De Bruin, Parker, and Fischhoff [31] found a positive correlation between age and calibration on an overconfidence/underconfidence probability judgment task using questions from *Complete Idiot’s* guides (in the task, participants had to give an answer and an estimation of the likelihood that their answer was correct). Lin, Zabrucky, and Moore [32] found that younger (mean age: 27 years) and older adults (mean age: 70 years) were equally well calibrated in predicting performance on a reading comprehension task.

A number of studies have reported age-related differences in retrospective metamemory and metaperception such that older adults are not as well calibrated as younger adults [33–36]. For example, Palmer et al. [36] found a negative relationship between age and calibration in a visual perceptual task. Additionally, a number of authors have hypothesised that metacognitive judgements are inaccurate in older adults when they rely on retrieval from memory [37]. Dodson and Krueger [33] found that older individuals (60–77 years old) were less well calibrated in tasks that required recalling newly learned materials (e.g. source identification and cue-recall tests), rather than general information. Finally, in memory tasks where participants are asked to prospectively predict performance when recalling a list of words, participants show weaker correlations between performance and their prospective predictions when compared to their retrospective evaluations [38]. This pattern is somewhat amplified in the older adults: their differences between predicted and actual scores in general are higher than the younger adults’ differences, with older adults predicting more than they actually recall.

Thus, there is a complex relationship between age and metacognition. There are somewhat equivocal findings, but a clearer pattern emerges, albeit limited to findings from research in metacognition in memory, if one considers prospective judgements in isolation. Here there is some suggestion that metacognitive accuracy relies on adequate memory function, and that inaccurate prospective judgements are caused by a failure to update expectations in performance (see also [39]) or the inappropriate use of generalised ‘rules of thumb’ such as midpoint anchoring, which are no longer applicable (e.g. [38]). Furthermore, research on running has indicated that as long as older age is not accompanied by a decline in physical capacities (e.g. VO2MAX) it can improve performance in long-distance races as a result of increased experience [29, 30]. Based on such findings, there is a clear role of experience in age; if older people have experienced, well-encoded and stored their previous race performance, whilst their capacity to perform remains the same, they should be able to make accurate evaluations of their performance. Otherwise, uninformed inferences about performance which are not based on feedback from performance or which ignore feedback from performance deterioration, might be inaccurate, out-of-date, and as with memory performance, over-estimations of performance.

In summary, we investigated the effects of key indicators of experience, gender and age on performance prediction in an annual half marathon. We did this using publicly available finish-time and demographic data, which we combined with predicted finish times that runners had submitted to the organisers at registration. Predicted finish times are used by race organisers to anticipate the flow of runners through various sections of the race, culminating in the finish funnel. Given the logistical insight they can offer, they are particularly beneficial to the organisation of large mass participation races, and are routinely collected at these events. The race in question was the mass-participation Alloa Half Marathon (with data from 2009 to 2015, excluding 2010, for which no prediction data were available), a prominent event in the Scottish athletic calendar for both competitive and recreational athletes. The aim of the present study was to identify variables that influence running calibration in order to provide trainers and race organisers with a better understanding of the processes that affect performance awareness as measured using calibration.

Based on the existing literature on performance prediction, and supplemented by findings from the psychological metacognition literature, we expected club affiliated runners to be better calibrated than unaffiliated runners, as an indirect indication of experience. Similarly, we anticipated runners who had completed the race multiple times to be more accurate in their predictions in their later, as opposed to their earlier, races. Although we anticipated overconfidence in general, we anticipated that women would be better calibrated than men as a result of relatively lower overconfidence. Finally, we investigated, but made no predictions regarding the relationship between age and performance calibration.

## Methods

### Data acquisition

The data used in the analyses presented here were gathered from two sources, the publicly accessible results documents released after each race, and the registration information gathered by the Alloa Round Table organisation that organises the Alloa Half Marathon 13.1 mile road race. This race is held in and around the town of Alloa in Clackmannanshire, Scotland and, taking place in March, is one of the first road half marathons in the annual road running season. It is relative flat (~320ft elevation gain) and not unduly affected by the weather. It attracts a high quality national field, with many winners who have represented or go on to represent Great Britain, typically running the race in under 80 mins (female) and 70 mins (male). Despite the high quality top end, the majority of the athletes who participate are club and hobby runners. As such, the race provides an excellent insight into how runners of all abilities prospectively assess their performances. Registration information for six races was obtained by negotiation with the Alloa Round Table—from the years 2009 to 2015, excluding 2010. These data were used in accordance with the Section 33 Exemption in the UK Data Protection Act which allows for personal data to be used by universities for research purposes as long as they are not used to make any decisions about an individual. We secured ethical approval for these analyses from the University of St Andrews Teaching and Research Ethics Committee (approval number: PS11442)

It was necessary to combine the data as the registration information contained the performance predictions, and the results contained the finish time performance data. The full set of fields in each source of data can be found in Table 1.

**Table 1:** Data fields and their sources.

Field	Data Type	Registration	Results
Name	Text	X	X
Sex	Categorical (“M”/“F”)	X	X
Club	Text	X	X
Date of Birth	Date (DD/MM/YY)	X	X
Prediction	Categorical (no prediction, “under 1:30:00”, “1:30:00 to 1:45:00”, “1:45:00 to 2:00:00”, “2:00:00 to 2:15:00”, 2:15:00 to 2:30:00”, “over 2:30:00”)	X	
Finish Position	Integer		X
Finish Gun Time	Time (HH:MM:SS)		X
Finish Chip Time	Time (HH:MM:SS)		X

**Note**: Elite, invited, and a small number of runners completing the registration form did not provide a prediction. These runners were excluded from all subsequent analyses. In the years, 2013, 2014 and 2015, runners were given the option of predicting “under 1:10:00”, “1:10:00 to 1:20:00” and “1:20:00 to 1:30:00”. To ensure consistency across all years, these categories were collapsed into “under 1:30:00” in the final dataset. Finish Gun Time is the time elapsed between the starter starting the race and the runner crossing the finish mat. Finish Chip Time is the time elapsed between the runner crossing the start (which in most cases is a number of seconds after the started has started the race) and finish mats. Finish Chip Time is therefore the more accurate measure of the time taken to run the race.

### Data processing

In later analyses, we examine within-subjects changes in performance and predictions across the six events for which we had data. By matching name, birthdate and demographic characteristics across races, we identified 1393 runners who had run the race more than once (888 two times, 321 three times, 124 four times, 46 five times and 14 six times). Our analyses focused on the runners who had completed the race at least three times—505 runners, of whom 488 runners produced analysable data across all three races (where runners had completed four or more races, we chose to look at the first three races, chronologically).

### Data analysis

Our analysis strategy was to use correlations for continuous variables such as age, and to use ANOVAs for categorical groups (such as gender or club membership). With such a large sample size, we paid particular attention to effect size and calculated partial eta squared and report Cohen’s d for the differences between means of performance. For correlations, we used a value of r = .20 when deciding what was a meaningful correlation. For calibration data, we examined whether the predicted intervals matched the observed intervals by using chi-squared tests to assess matched, underestimated, and overestimated predictions between groups. Cramer’s V values were calculated to assess effect size.

## Results

We first present performance (i.e. the Finish Chip Time variable) and predicted performance (i.e. the Prediction variable) according to a number of key demographics. Next, we examine the effects of runner demographics on calibration. Finally, we present the effects of race experience on performance and calibration. Because of missing data, there are differences in the numbers contributing to each analysis. These differences are reflected in the samples sizes noted for each analysis.

### Performance

**Fig 1:**
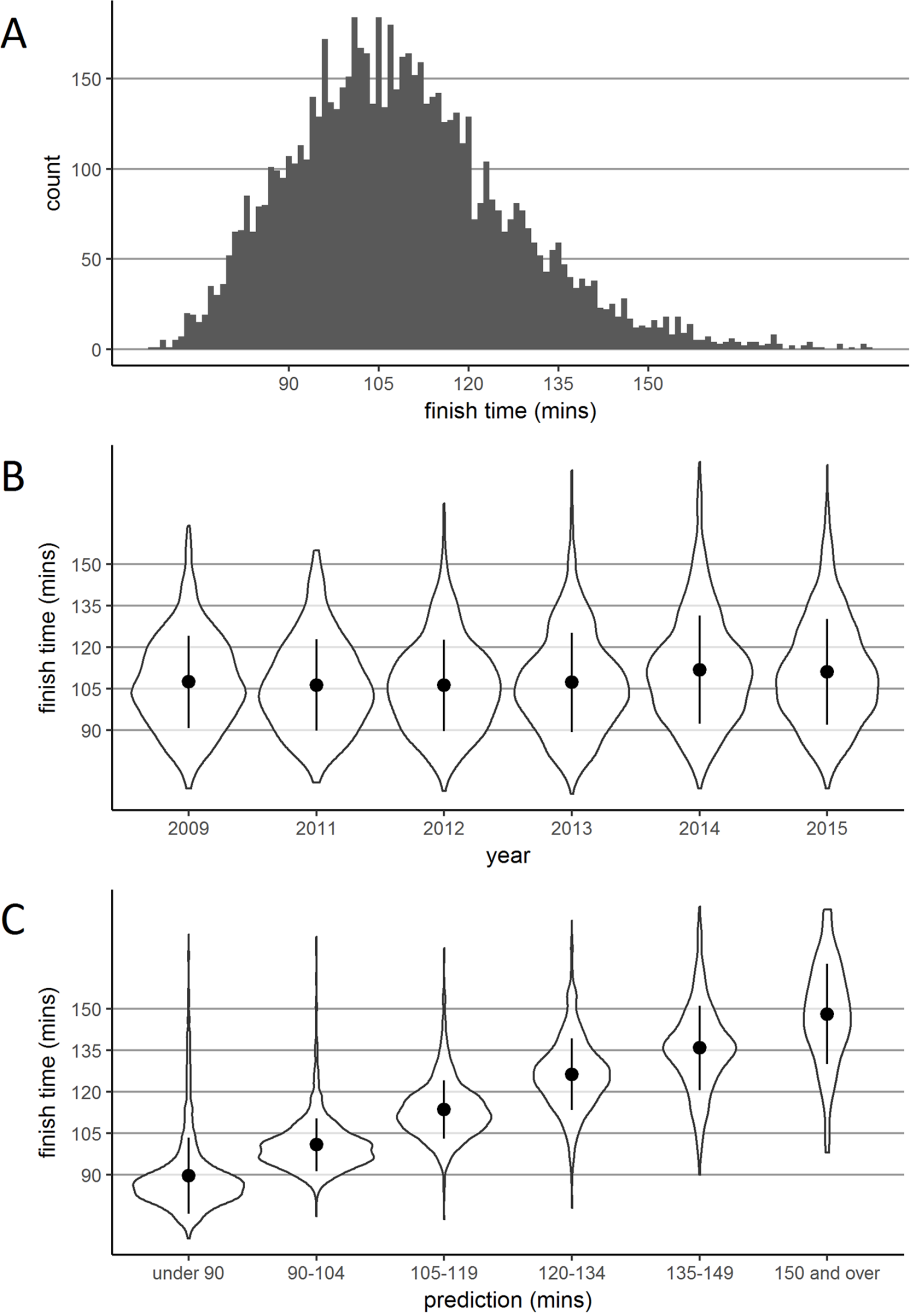
Finish times and densities according to year and prediction. Panel A shows a histogram of finish times collapsed across all years. Panel B shows a violin plot of finish time density according to year. Panel C shows a violin plot of finish time density according to the prediction window selected at registration. In Panels B and C, the perimeter of each plot illustrates density, the central point represents the mean, and the vertical line represents +/- one standard deviation.

Collapsed across all years, the time taken to complete the Alloa Half Marathon (in minutes, rounded down) is shown in Fig 1A. The mean time taken to complete the race was 108 min and 54 sec (SD = 18.2, *n* = 7018). To use as a point of reference, the world record for men is 58 min and 14 sec and for women is 65 min and 24 sec. Hereafter all performance units are minutes.

#### Performance by year

Fig 1B presents violin plots showing finish time density across the six years of races—this visualisation controls for increasing numbers over the years. Mean (SD, *n*) finishing times were as follows: 2009–107.4 (16.7, 844); 2011–106.3 (16.5, 822); 2012–106.2 (16.6, 1020); 2013–107.3 (18.0, 1196); 2014–111.8 (19.5, 1508); 2015–111.1 (19.1, 1628). Interestingly, there was a significant change over time, *F*(5,7012) = 23.57, *p* < .001, *η*_*p*_^*2*^ = .017, such that the 2014 and 2015 races were completed significantly slower than all previous races (all *p*s < .001)—no other pairwise comparisons revealed significant differences (all *p*s > .999).

#### Performance by prediction

Fig 1C presents the finishing time density according to predicted finishing time. Mean (SD, *n*) finishing times for each prediction window were as follows: ‘under 90’, 89.7 (13.7, 1327); ‘90–104’, 100.8 (9.5, 1932); ‘105–119’, 113.6 (10.5, 2373); ‘120–134’, 126.3 (13.0, 926); ‘135–149’, 135.8 (15.3, 356); ‘150 and over’, 148.1 (18.1, 104). Participant performance was largely in line with predictions, with finish time distributions cascading up according to the “expected finish time” window selected at registration.

#### Performance by experience (club membership)

**Fig 2:**
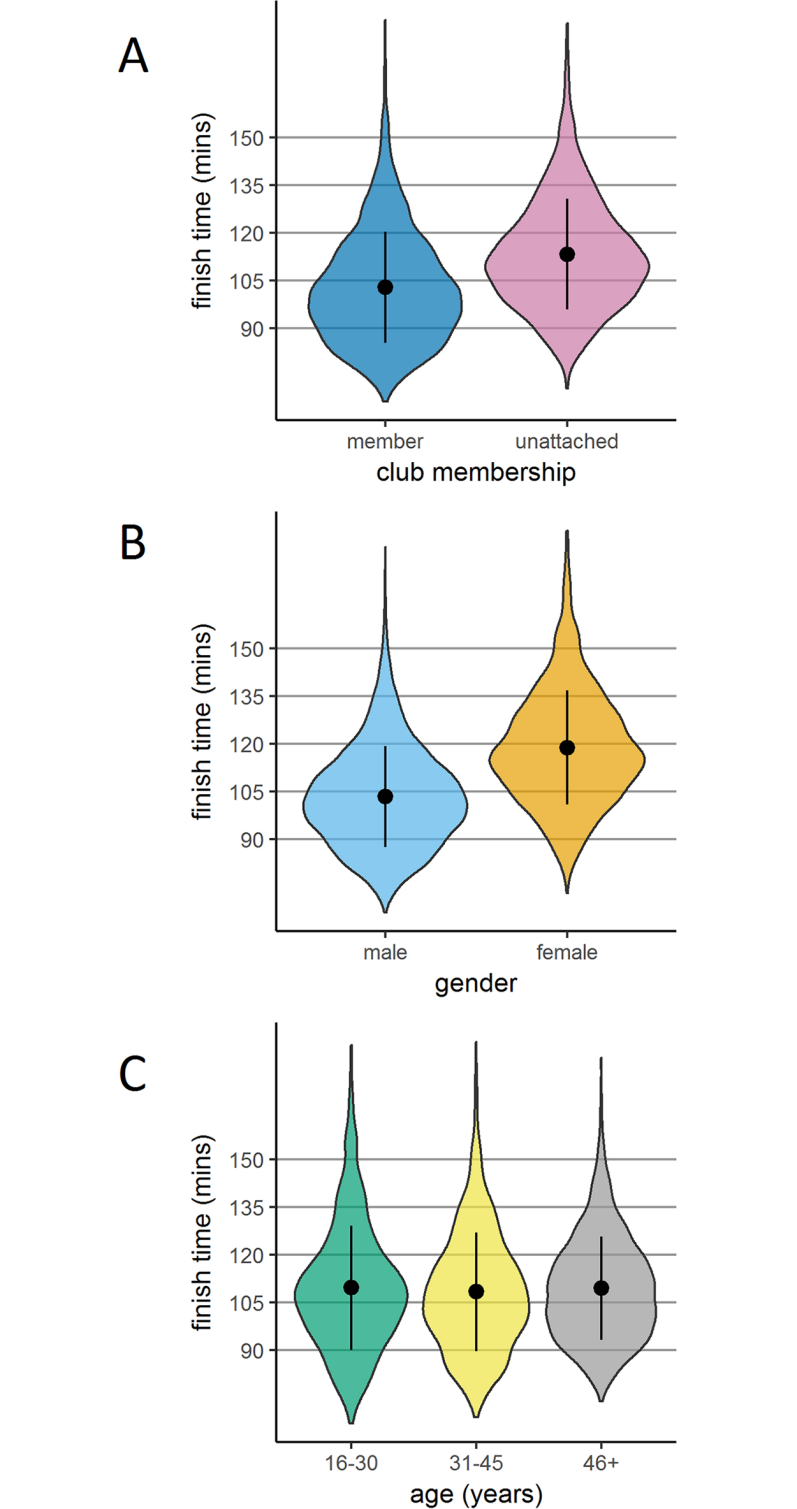
Finish times according gender, age and club membership. Violin plots of finish time density according to club membership (Panel A), gender (Panel B) and age category (Panel C; ‘46+’ is runners aged 46 and older). The perimeter of each plot illustrates density, the central point represents the mean, and the vertical line represents +/- one standard deviation.

**Fig 3:**
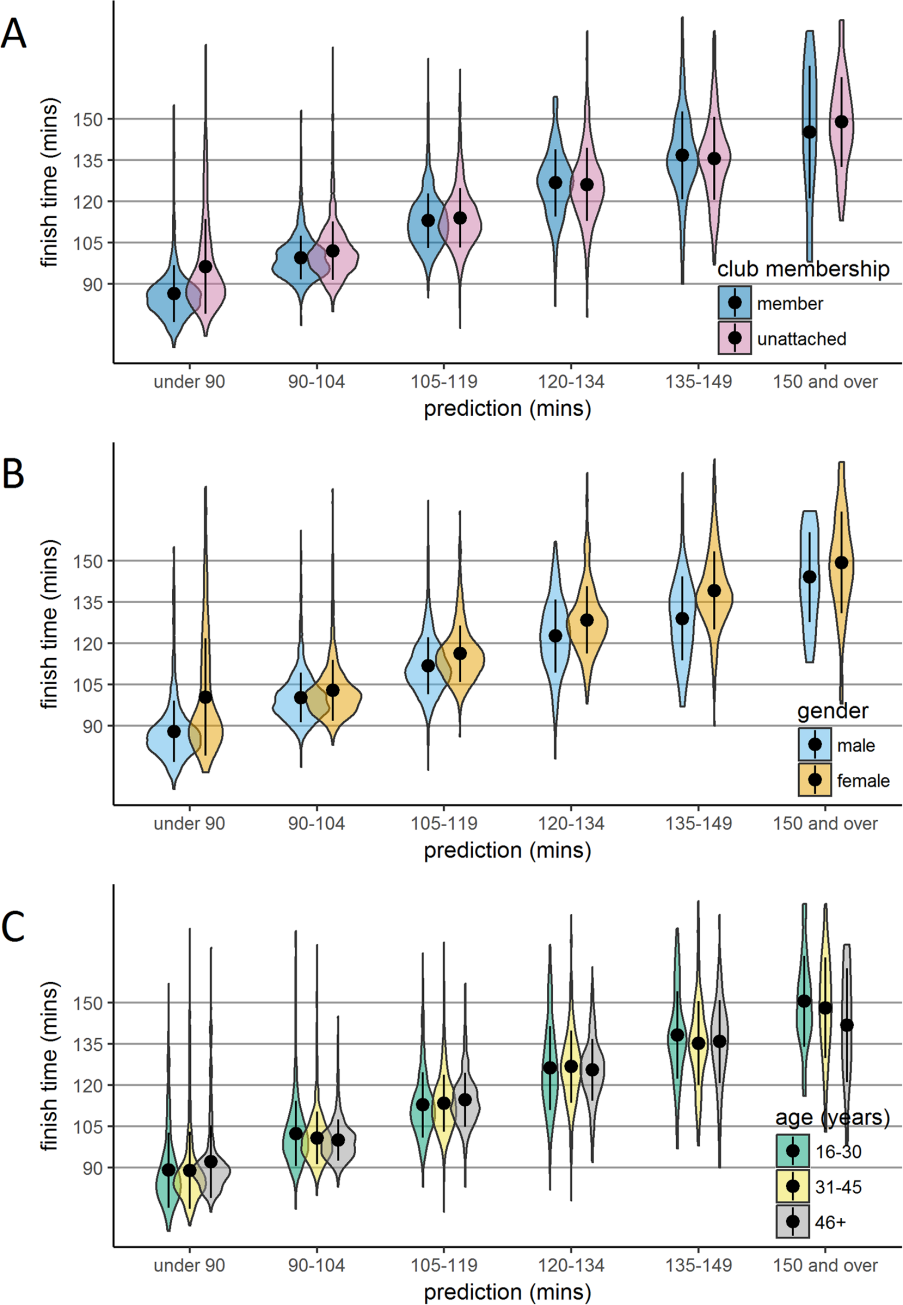
Finish times according to the interaction between demographics and prediction. Violin plots of finish time density according to prediction and club membership (Panel A), gender (Panel B), and age category (Panel C). The perimeter of each plot illustrates density, the central point represents the mean, and the vertical line represents +/- one standard deviation.

Fig 2A presents finish time density according to club membership (there were 2950 who declared a club affiliation and 4068 runners who were unattached to a club at the time of registration). Unattached runners were slower to finish than club runners (113.3 [17.4] compared to 102.8 [17.5], *t*(6562.7) = 25.55, *p* < .001, *d* = .602. Fig 3A shows that this performance advantage for club runners was driven by faster performance in runners making predictions at the extremes (‘under 90’ and ‘150 and over’).

#### Performance by gender

Fig 2B presents finishing time density according to gender (there were 4512 males and 2491 females). Men completed the race in a shorter mean time than women: 103.4 (SD = 15.9) compared to 118.8 (SD = 18.0), *t*(4712.7) = 35.61, *p* < .001, *d* = .909. Fig 3B shows that this difference between men and women in performance held across all prediction windows. The only group mean performance which was slower than predicted was for women making ‘under 90’ predictions.

#### Performance by age

**Fig 4:**
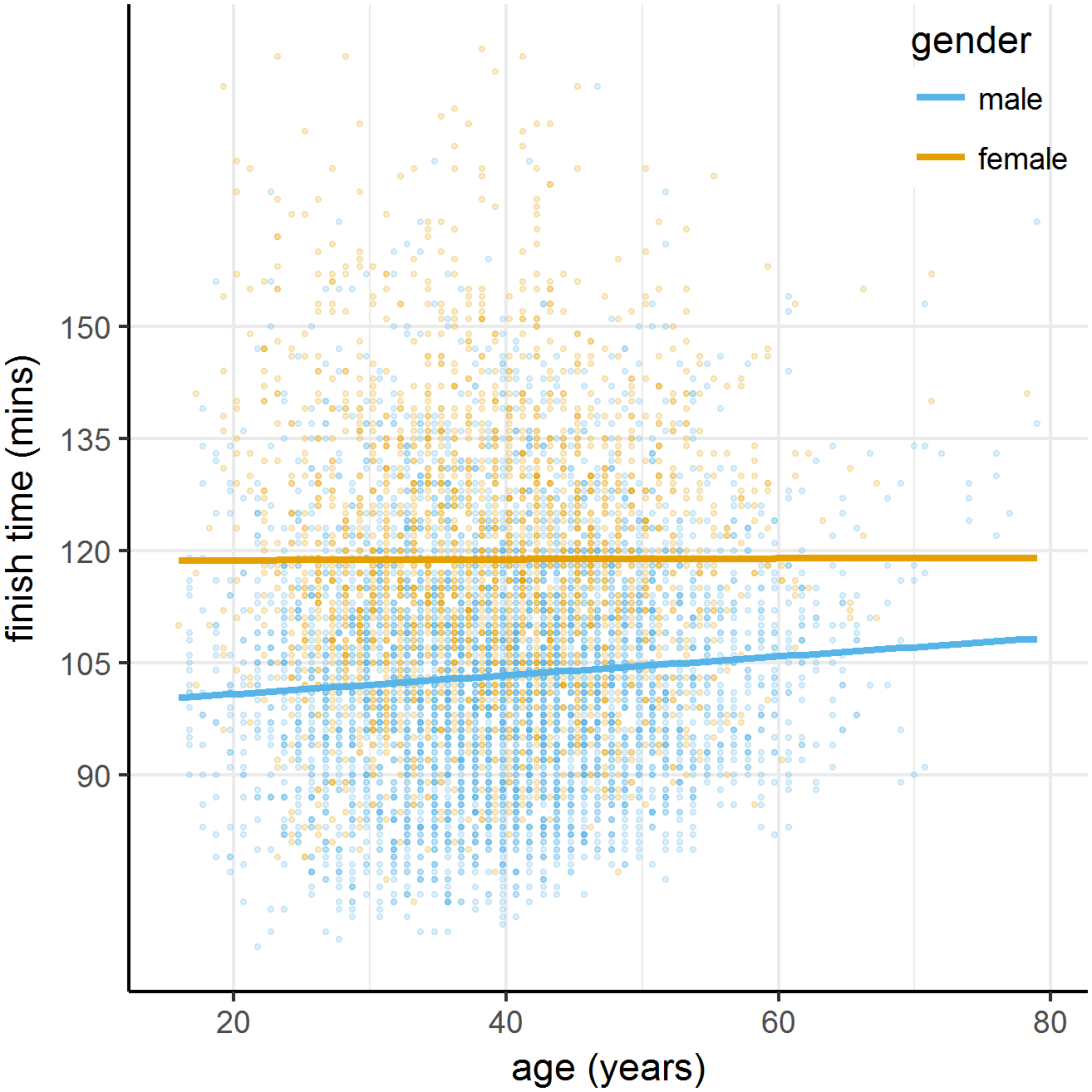
Scatterplot showing age against finish time. Males (blue) and females (yellow) are plotted with alongside their lines of best fit.

We first treated age as a continuous variable to examine the correlation between age and performance. Fig 4A shows a scatterplot of age against finish time. In the entire sample, there was no correlation between age and performance, *r*(7001) = .011, *p* = .349.

We also coded age as a categorical variable with three categories, shown in Fig 2C. Mean (SD, *n*) finishing times differed significantly as follows: ‘16–30 years’, 109.6 (19.6, 1255), ‘31–45 years’, 108.4 (18.7, 3883); and ‘46 years and over’, 109.4 (16.2, 1865); *F*(2,7182) = 3.756, *p* = .023, *η*_*p*_^*2*^ = .001. Age compressed performance, such that there were fewer runners in the ‘46 years and over’ group finishing at the extremes. Importantly, age alone did not lead to slower performance. Indeed, examination of Fig 3C shows that increased age is even associated with faster performance in runners making slower predictions (‘135–149’ and ‘150 and over’).

### Predicted performance

**Fig 5:**
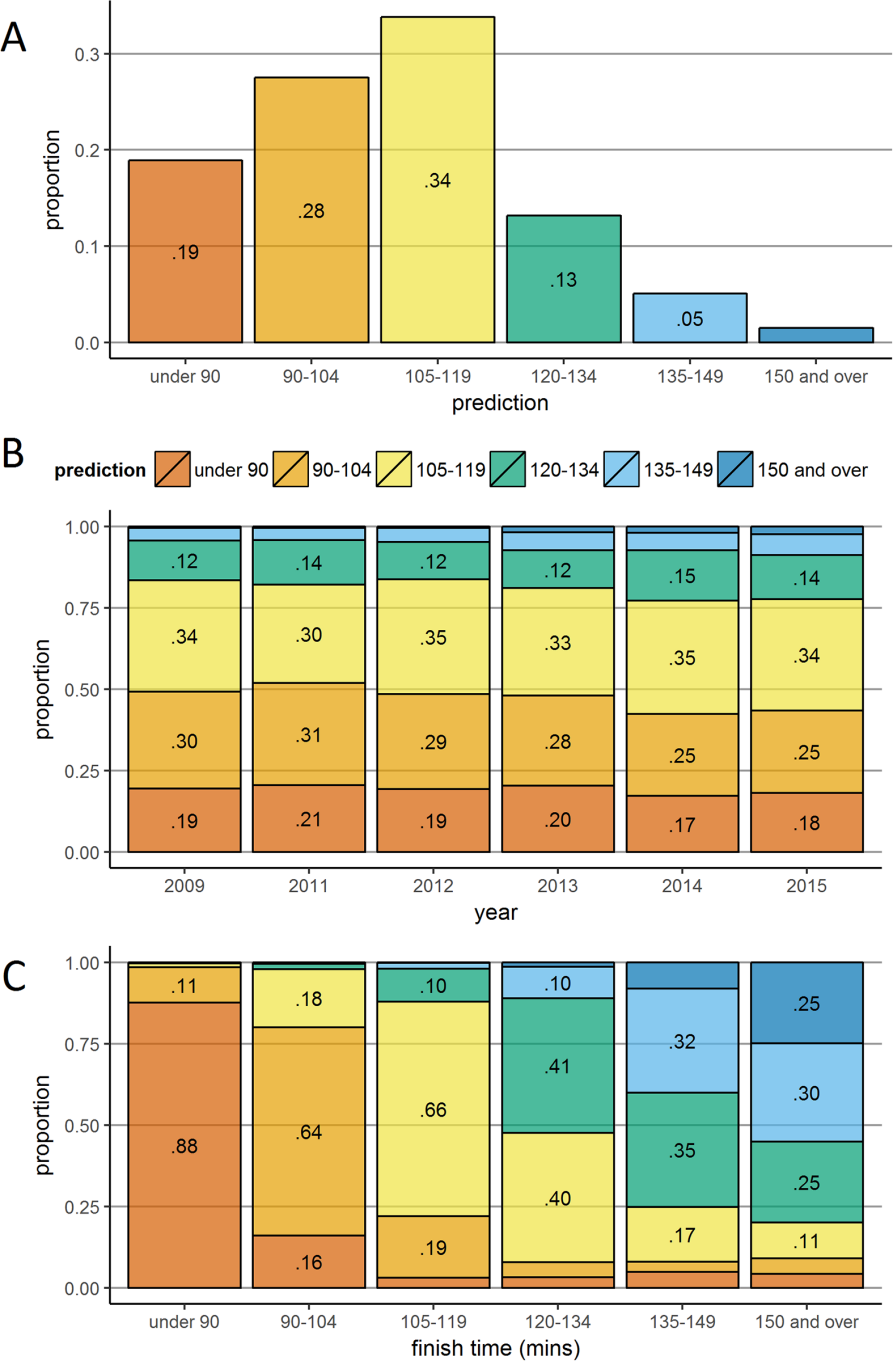
Predictions according to year and finish time. Panel A shows the proportion of predictions collapsed across all years. Panels B and C shows stacked bar plots of predictions according to year and eventual finish time respectively. Prediction proportions less than .05 (Panel A) and .10 (Panels B and C) are not labelled.

Collapsed across all years, the proportions of predicted times are shown in Fig 5A. The most frequently made prediction was 105–119 minutes (34% of all runners) and the least frequently made prediction was 150 minutes and over (1% of all runners).

#### Predicted performance by year

Fig 5B presents prediction proportions by race year, with no marked differences across races. In all subsequent analyses, we collapse across years.

#### Predicted performance by performance

Fig 5C presents prediction proportions according to actual finish times. Across all finish times, the corresponding prediction window is represented well above the overall base rate shown in Fig 5A (e.g. 88% of those finishing in under 90 minutes chose this prediction window [19% base rate], 25% of those finishing in over 150 minutes chose this prediction window [1% base rate]). Complementing the relationship observed when finish time data were binned by prediction (Fig 1C), prediction data binned by finish time showed strong correspondence, though this began to break down in the 120–134 minute window and beyond.

#### Predicted performance by experience (club membership)

**Fig 6:**
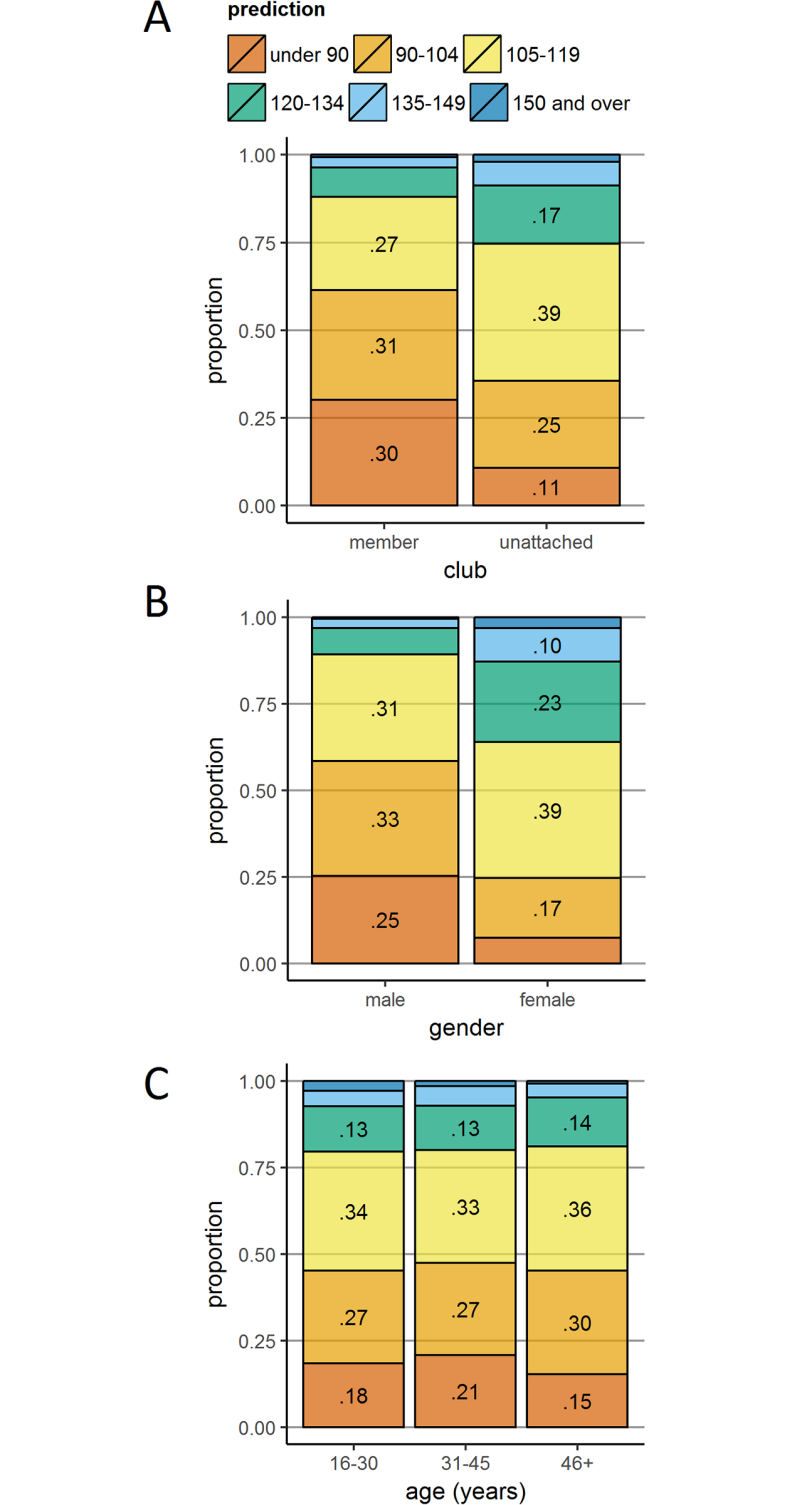
Predictions according to club membership, gender and age. Stacked bar plots of predictions according to club membership (Panel A), gender (Panel B), and age category (Panel C; ‘46+’ is runners aged 46 and older. Prediction proportions less than .1 are not labelled.

The previously presented differences in performance, club runners completing the race faster than unattached runners, were echoed in the predictions made by the two groups. Fig 6A shows that club runners made significantly greater use of the faster prediction windows than unattached runners, *χ*^*2*^(5) = 605.93, *p* < .001, *V* = .131.

#### Predicted performance by gender

**Fig 7:**
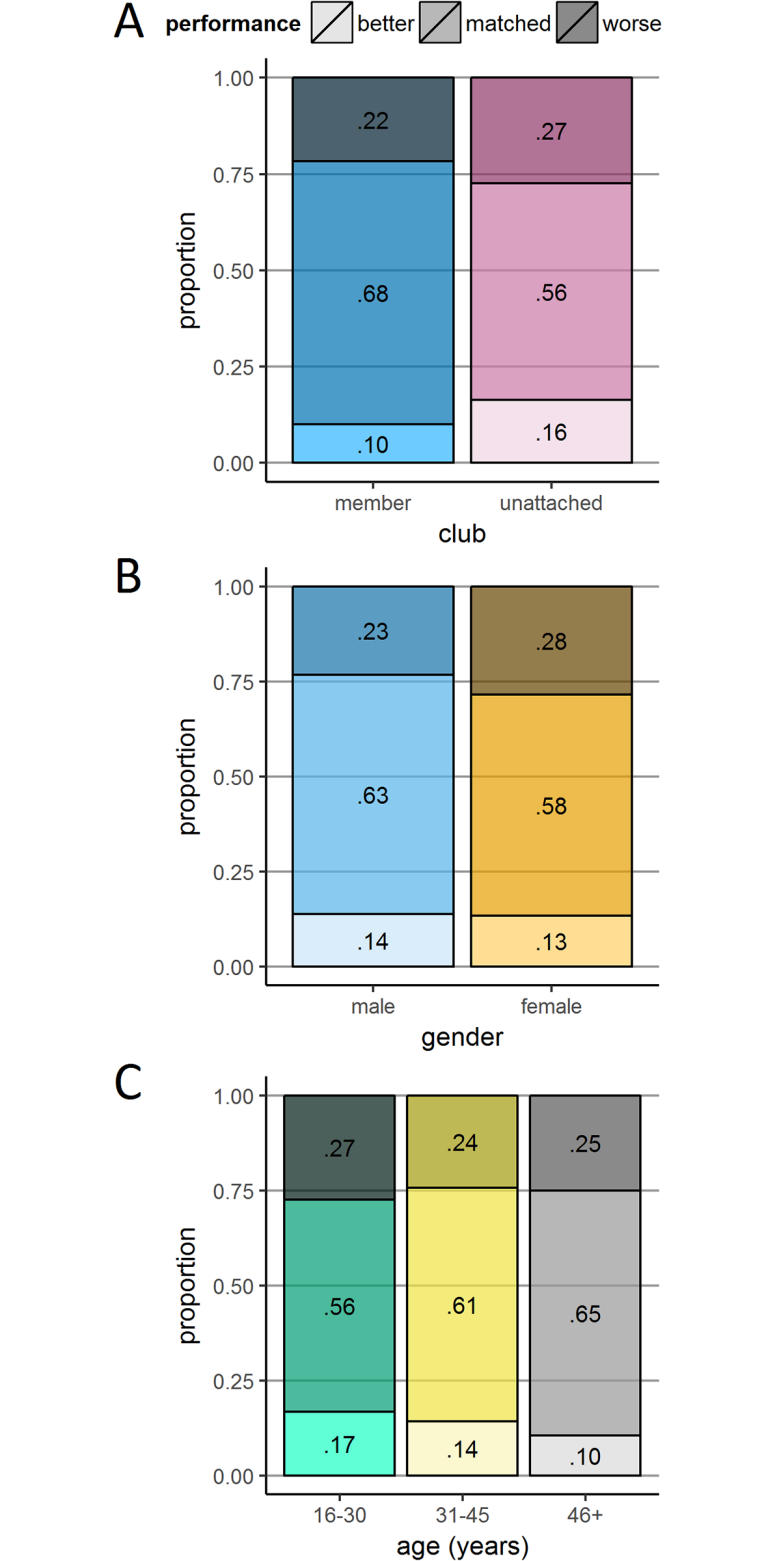
Prediction discrepancies according gender, age and club membership. Three panels plotting the proportions of runners whose performance was better (faster) than predicted; matched their predictions; and was worse (slower) than predicted. The demographic variables used to split runners were: club membership (Panel A); gender (Panel B); and age category (Panel C).

Fig 6B illustrates the pattern of predictions according to gender, such that women tended to predict significantly slower finish times than men, *χ*^*2*^(5) = 984.08, *p* < .001, *V* = .167. The most widely chosen prediction windows by men (90–104 minutes; 33%) and women (105–119 minutes; 39%) reflect the previously presented finding that men’s finish times were approximately 15 minutes faster than women’s finish times. Fig 7A shows that the predictions made by men and women were largely in line with their finish times.

#### Predicted performance by age

Fig 6C shows that there were slight differences in prediction proportions across the younger two groups, with a significant expansion of the middle predictions (and correspondingly fewer extreme predictions) in the ‘46 years and older’ group, *χ*^*2*^(10) = 61.16, *p* < .001, *V* = .030.

### Calibration

**Fig 8:**
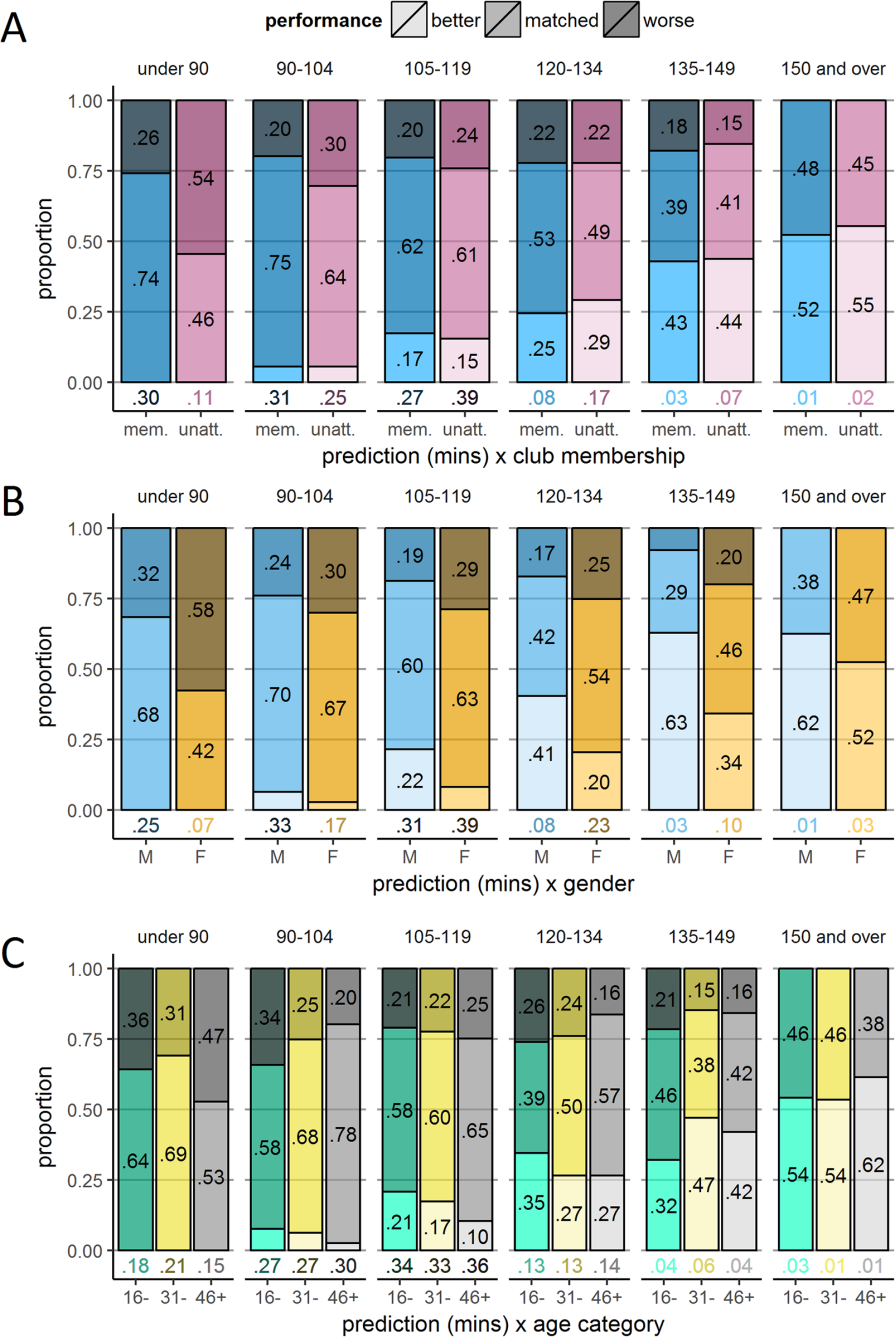
Performance according to the interaction between demographics and prediction. Panels A, B, and C show stacked bar plots plotting categorical discrepancy—the proportions of runners whose performance were better (faster) than predicted; matched their predictions; and were worse (slower) than predicted. The independent variables used to organise data are club membership (Panel A), gender (Panel B), and age category (Panel C), and how they interact with prediction times.

Our approach within this section is to examine calibration using a categorical measure with three performance outcomes: better (faster performance than predicted); matched (performance within the predicted window—perfect calibration); worse (slower performance than predicted). Fig 7 presents calibration using stacked bar charts. For the whole sample, performance relative to prediction was as follows: better 13.7%; matched 61.3%; worse 25.1%. These findings indicate that participants were overall more likely to overestimate than underestimate their performance, although they were, on average, very well calibrated given the bins used. Below, we present calibration according to our three key demographic variables. For a finer granularity, we also bin these data according to prediction window (Fig 8). Once again, for these analyses we collapse across race year.

#### Calibration by experience (club membership)

Fig 7A plots prediction accuracy by club membership. In accordance with our hypothesis of experience-led calibration improvement, the likelihood of the prediction matching the performance was lower in unattached runners than club runners (56.2% compared to 68.3%), *χ*^*2*^(2) = 113.82, *p* < .001, *V* = .127. Compared to club runners, unattached runners were more likely to perform both better (16.3% compared to 10.0%) and worse than predicted (27.5% compared to 21.7%). Therefore, there was no systematic under- or over-confidence in the unattached runners, they were just more inaccurate than club runners overall. Fig 8A shows that club runners were better able to match their predictions, particularly over the two fastest prediction windows.

#### Calibration by gender

As shown in Fig 7B, predictions by females were less likely to match their performance compared to males (58.1% compared to 63.0%), *χ*^*2*^(2) = 24.45, *p* < .001, *V* = .059. Cases where performance was better than predicted were comparable across females (13.4%) and males (13.8%), leaving the bulk of the difference in matched performance outcomes driven by a greater tendency for females to perform worse than predicted (28.5%) compared to males (23.2%) going against our prediction of higher male overconfidence. Fig 8B shows that males’ relative tendency to perform better than predicted was consistent across all prediction windows.

#### Calibration by age

Coding age according to the three previously defined age categories, Fig 7C indicates that runners with perfect calibration increased with age (‘16–30 years’, 55.9%; ‘31–45 years’, 61.4%; ‘46 years and over’, 64.6%), *χ*^*2*^(4) = 36.21, *p* < .001, *V* = .036. This slight increase in calibration accuracy was associated with decreases in both performance exceeding prediction (‘16–30’, 16.7%; ‘31–45’, 14.2%; ‘46 and over’, 10.5%) and falling short of prediction (‘16–30’, 27.3%; ‘31–45’, 24.3%; ‘46 and over’, 25.0%). Across prediction windows (Fig 8C), age had a similar effect on calibration as it did on performance, yielding apparent differences only in the fastest and slowest prediction windows. For the ‘under 90’ prediction category, those in the oldest age category underestimated running times relative to younger runners, whereas for the ‘150 and over’ category, they overestimated relative to younger runners.

### Race experience

There were 488 runners who both made a prediction and ran the race at least three times. We examined performance and prediction accuracy the three races run by this subsample of runners. It should be noted that the race repetitions presented are not necessarily taken from the same years, but are drawn from any of the years for which we have data and presented in chronological order (e.g. runner X’s three repetitions could be 2009, 2011, 2012, whereas runner Y’s could be 2011, 2013, 2015).

#### Race experience and performance

**Fig 9:**
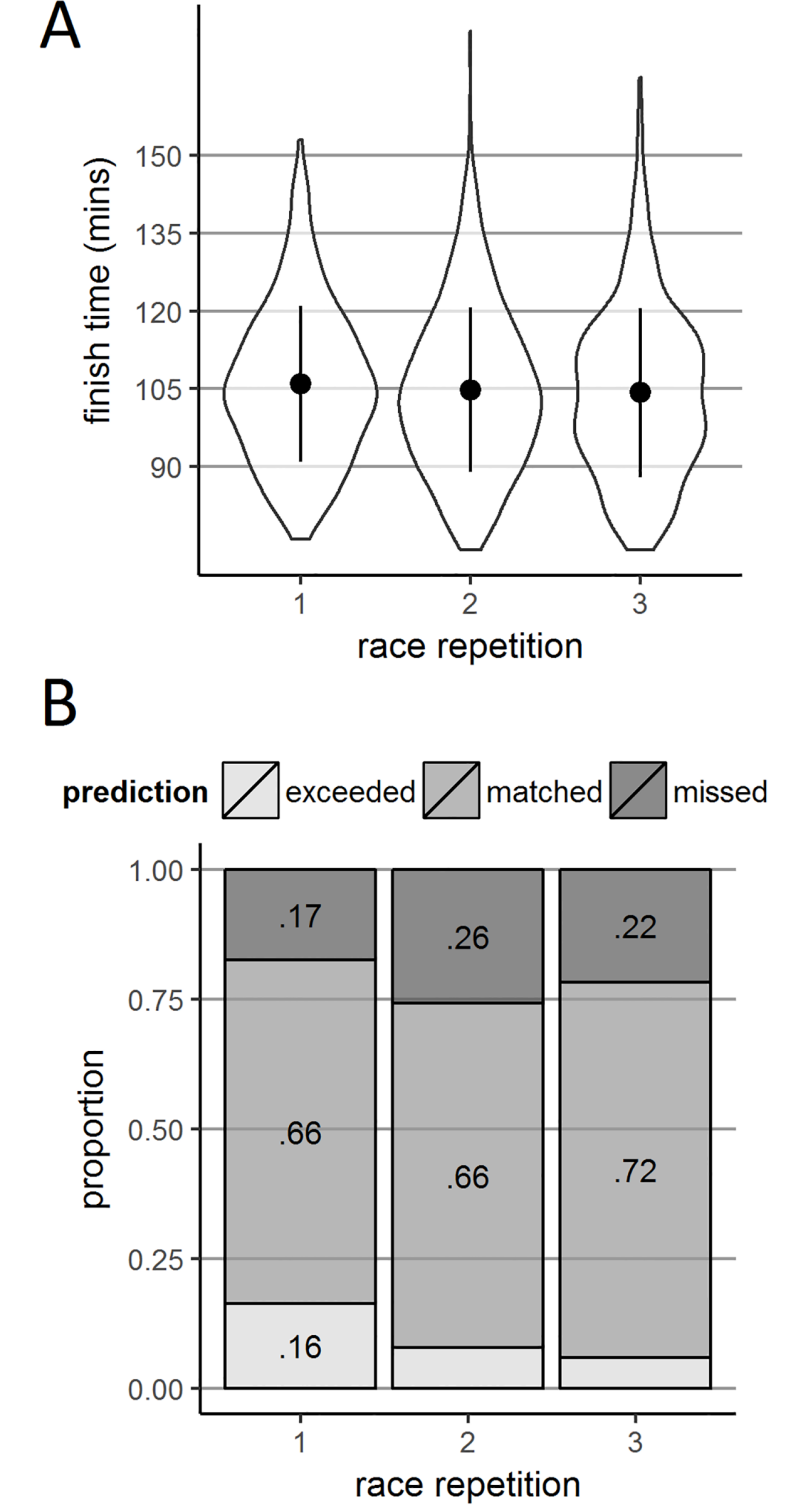
Finish times and prediction discrepancies according race repetition. Panel A shows violin plots of finish time density according to race repetition. Panels B shows stack bar charts plotting categorical discrepancy according to race repetition. Panel C shows violin plots of continuous prediction discrepancy density according to race repetition. These are within subjects data, meaning that the same 483 runners who completed the race at least three times contribute to each violin or bar within the three panels.

Finish times shown in Fig 9A illustrate that the expectation of performance increasing with experience was borne out in the data. Mean (SD) finish times were as follows: race 1, 106.0 (15.0); race 2, 104.8 (15.9); and race 3, 104.3 (16.3). Over the course of running the race three times, runners’ times decreased significantly, *F*(2,974) = 8.41, *p* < .001, *η*_*p*_^2^ = .017, with significant differences between races 1 and 2 (*p* = .018) and races 1 and 3 (*p* = .001) but not races 2 and 3 (*p* = .498). On average, runners improved nearly 2 minutes over the three races.

#### Race experience and calibration

Fig 9B illustrates a trend towards increased accuracy with increasing race repetition, with the proportion of accurate predictions increasing by the third race (race 1, 66.2%; race 2, 66.4%; race 3, 72.3%), *χ*^*2*^(4) = 39.94, *p* < .001, *V* = .083. Interestingly though, the number of runners whose performance exceeded their predictions decreased with increasing race repetitions (race 1, 16.4%; race 2, 7.8%; race 3, 5.9%). Conversely, there was no clear pattern in the proportion of runners whose performance fell short of their predictions (race 1, 17.4%; race 2, 25.8%; race 3, 21.7%). In sum, these findings suggest that whilst runners who race multiple times become increasingly accurate in their predictions and they are also more likely to perform worse (rather than better) than they predict.

## Discussion

We investigated performance prediction calibration in runners who participated in the Alloa Half Marathon. We found that runners’ predictions and race times were mostly matched, indicating an overall tendency towards good calibration. Of those who did not accurately predict their times, runners were more likely to overestimate rather than underestimate their performance. Breaking our data down according to key variables, we found that experience, measured indirectly through club membership and directly through race repetition, led to an increase in calibration. Note that it is not known whether our non-members running the race for the first time were naïve—it is possible, even likely, that they had competed other similar races before. Gender and age also produced surprising results. Women were slightly less well calibrated than men, and were also more likely to overestimate performance than men. Aging was associated with slightly better calibration—older runners were more accurate in their predictions than younger runners. We now discuss each of these findings in turn.

### Experience

We predicted that both direct and indirect markers of experience would be linked to higher calibration in more experienced runners. Bearing this out, club-affiliated runners were significantly more accurate in their predictions than unaffiliated runners. Beyond speculation that this sample of club runners were more likely to have been runners for longer (and therefore to have had more opportunity to develop their calibration from their own experiences), club membership puts runners in the position of regularly receiving feedback from coaches and other club members, giving them further opportunity to reflect on their performance and better understand their running capacity. Although confounded by a number of unmeasured factors that could have influenced calibration (e.g. type of club affiliated with, presence of coach, programme of training, number of club sessions per week, and place of training), these data show that membership of a running club is associated with increased understanding of one’s own likely performance in races.

The beneficial effect of experience on calibration was also found in our analysis of predictions across race repetition. Runners who had participated in the race before were better able to predict their performance in their third race than in the first two. Compared to the first race, races two and three showed greater likelihood of yielding performance that fell short of predictions, with the overall benefit to calibration resulting from greatly reduced likelihood of performance exceeding predictions. In something of a paradox, although runners became better generally calibrated with race experience, they were also more likely to be overly optimistic in their predictions. Without additional data, it is difficult to ascertain why this may be the case, though it may be that runners repeating the race are more motivated to perform better than their previous race, so make more ambitious predictions than for previous races. This raises the issue of how runners view the predictions they make at registration. We have so far considered them as relatively pure metacognitive windows into anticipated running performance, but they could alternatively serve a number of different purposes, discussed below.

### Gender

Surprisingly, we found men to be *better* calibrated than women. Whereas both genders made prediction that were exceeded by performance to a similar level, women showed higher overconfidence, being more likely to predict faster performance than was achieved. As outlined in the Introduction, higher overconfidence is typically observed in men [40], whilst women are more likely to reflect on their cognitive performance [26]. The present findings on gender are difficult to explain, as they go against previous evidence on gender differences in confidence. Furthermore, given the large magnitude of the sample size used and the broad range of runners participating, it is unlikely that the differences between the literature and our results could be attributed to such factors. A potential explanation for our unexpected findings could be given in terms of the ways in which the two genders gave predictions. When asked to predict performance, participants were not provided with further specifications on how they should make their predictions. It is possible that gender differences arose then, potentially leading to women predicting their ideal/goal performance and men giving realistic predictions. Krouse, Ransdell, Lucas, and Pritchard [41] used questionnaires to examine traits of female runners, which suggested that women are not motivated by competitiveness in running, but health, psychological coping, and personal achievement instead. Furthermore, they reported to be more likely to set task-specific goals (e.g. for a specific race) as opposed to having global expectations. It is thus possible that women *hoped* to perform as well as they predicted, whereas men *expected* to perform according to their predictions. However, this speculative explanation needs to be tested. Krouse et al.’s study was descriptive, i.e., did not use inferential statistics for their analysis, and made no comparisons between men and women. Consequently, it is imperative for future research to investigate how men and women make predictions when presented with no further instructions, and whether the present differences still hold when athletes are asked to give realistic expectations of their performance as opposed to what they hope to achieve.

### Age

We found a positive relationship between age and running calibration—younger runners were less well calibrated than middle-aged runners, who were in turn less well calibrated than older runners, even though the groups only differed to an inconsequential degree in terms of performance. These findings run counter to cognitive research which trends towards observing reduced metamemory and metaperception calibration with age [33–36]. As opposed to perception and cognition, which are faculties exercised in a number of forms on a continual basis, running is an activity that must be voluntarily engaged in—people do not tend to opt out of cognition or perception but often decide that they have become too old to run [42]. As such, the older runners in this sample are likely not just more experienced runners, they are also runners who have, for psychological, lifestyle and a lack of physiologically limiting reasons, continued to engage in running and racing. Moreover, race times are concrete values which will presumably be relatively easily stored and incorporated into self-representations. It would thus be of interest to see how novice or first-time older runners predict their performance.

There are some difficulties in interpreting this positive finding, not least the difference in ages between this sample and those considered by metacognitive studies as older (e.g. 60–80 years old [33, 34, 40]). Nonetheless, these data suggest that those who continue to run over their lifespan, or who come to it at a later age, are slightly better calibrated than those with less experience, or who start young. We were not in a position to collect data beyond those presented here. The number of previous races completed, years training amassed, measures of cognition, physical health, and motivation would all have helped tease apart age, experience and a range of other factors as underpinning this age effect. It would be relatively straightforward to include a small number of additional questions at registration that would serve this purpose, and we suggest that this would help add context to the current finding.

### Future directions

Our study was a first attempt to examine metacognitive patterns in the predictions of runners engaged in races and derived simply from a few questions asked during registration. Further research should examine the training programmes runners used or the weekly mileage they logged in preparation for the race. It goes without saying that differences in preparation affect race performance, with key findings that interval or fast-paced training leads to faster finish times compared to training at a constant pace for a longer period of time [4, 5]. More relevant to our interests though, are the potential effects that training regimes could have on performance calibration. It is conceivable that runners who have experience of training at race pace or using faster-than-race pace intervals could better know how they handle these situations, leading to more accurate predictions. Similarly, runners who prepare at a constant, slower pace might underestimate the demands of a higher race pace, leading to performance overestimation. Future research should consider these preparatory strategies and their effects not only on performance, but also on performance calibration. One interesting point is that a few studies show that making metacognitive evaluations increases cognitive performance (e.g. [43]). This is thought to be due to focussing the attention on the task in hand, but equally to increasing the depth of processing of the materials whilst the prospective evaluations are being made (e.g. [44–46]). If anything, this kind of effect, beating one’s predicted time, is likely to be stronger in racing, where the prediction may acts as a target as well as focusing the attention of the runner.

Although we used club membership and number of previous races completed as indicators of experience, we did not have access to complete data regarding previous experience. Past research has used previous personal best times in a race as a proxy of experience, finding them to be significant predictors of total race time in either the same or a different race [6–10]. The present study did not ask for previous personal best times in the Alloa Half Marathon, which could have further indicated the effect of experience on calibration. Nonetheless, it is not clear whether previous best times should be considered a measure of experience or competence. It is possible they can explain race time variation simply because they are a good indicator of an athlete’s performance capacity. It would thus be interesting to investigate both the extent to which personal best times at previous races represent experience or competence, as well as their effect on performance calibration. Furthermore, as outlined previously, age and experience share variance, making it difficult to attribute better calibration to one or other variable exclusively. This needs to be addressed by better controlling for experience, allowing for a more direct examination of the effects of age in running calibration.

Additionally, using club membership as a proxy for experience (and access to experience) is crude, especially when we consider the growing success of a range of running clubs in Scotland under the support of the Scottish Government-funded JogScotland organisation. These non-traditional jogging clubs are different from more established athletics clubs in that they promote and support jogging and running often as a healthy lifestyle intervention. It is conceivable that for many runners, JogScotland groups are a way into running (rather than a further step that is taken to consolidate one’s running, as is typically the case with traditional athletics clubs), making them a poor marker of experience. Finally, our within-subjects analysis used a very limited set of races, making it all the more compelling that calibration improvements were found. Given the huge number of races available for runners to enter, it is very unlikely that the race we considered to be each runner’s first, was in fact their first half marathon (or indeed their first Alloa Half Marathon). More likely, the calibration improvements we observe are evidence of the continuing benefit of iterative calibration refinements over increasing numbers of races.

Perhaps most crucially, the nature of the predictions made by the runners included in the sample was heterogeneous in a number of ways. Participants were not given guidance on how they should make a prediction (as a best/worst case, as a target, etc.), nor did participants give their predictions at a uniform time before to the race (some runners gave predictions months before, others, on the day of the race). It is conceivable that some groups of runners could have made aspirational predictions, some could have made realistic ones, some could have made unrealistic predictions for strategic reasons (e.g. under the misapprehension that there would be starting pens at the race), and some could simply have made a mistake. Similarly, we would expect runners matched on experience to make better predictions closer to the race, as they have a better idea of their own training and conditioning, and the conditions on the day. Any systematic variation in these factors would confound interpretations of performance calibration, meaning that the results remain very difficult to interpret. However, it appears that the results are not random, as there are clear systematic differences according to club membership, race repetition, gender, and age. Nonetheless, a more hands-on approach to collecting data, as opposed to the present opportunistic data analysis, could have dealt with these issues of interpretation and should be implemented in future research, though it is a strength of the data that in spite of these issues, a number of observations regarding performance prediction differences could be made. The sensitivity afforded by the large samples used allowed us to interrogate effects that could be having an influence on a population level.

### Practical applications

The present findings could have significant implications for coaches, athletes and running communities. Our data suggest that coaches should acknowledge the previous experience of runners and adapt their coaching style accordingly. For example, they may wish to guide less experienced runners through the process of setting performance goals and predictions in order to maintain realistic expectations and minimise the disappointment of setting a goal that is unlikely to be achieved. Similarly, if future research confirms that women are likely to set goals when asked to predict their performance, whilst men make more likely outcome predictions, then coaches could adapt their assistance of female and male athletes appropriately. Women may benefit from the process of setting race goals to motivate their training in ways that men do not. Finally, increased metacognitive awareness older adults show could be recognised in catering to their needs as athletes, and their roles within running communities. It is not inconceivable that all of these factors could be leveraged together such that specific groups within running clubs and communities could take some of the burden from individual coaches. Older, experienced runners with a knowledge of how they have previously anticipated their own performance (e.g. as a training goal) could contribute to a broader network of mentors capable of guiding less experienced runners through the same decisions as they make their way through their own running careers.

### Conclusion

Our investigation of the factors which affect running calibration using data from the Alloa Half Marathon provided us with several interesting findings. Experience as indicated indirectly by club membership or directly by number of previous races completed was accompanied by better calibration. Contrary to our expectations, women were less well calibrated than men, predicting faster finish times more frequently. Older age also appeared to serve as a proxy for experience: older runners were the best calibrated age group, followed by middle-age runners, with young runners the least well calibrated group. We intend for these findings to serve as a starting point for a further investigation of metacognitive processes in running, so as to better understand how runners monitor and control their performance.
